# Beyond Bio-Inspired Robotics: How Multi-Robot Systems Can Support Research on Collective Animal Behavior

**DOI:** 10.3389/frobt.2022.865414

**Published:** 2022-06-20

**Authors:** Nikolaj Horsevad, Hian Lee Kwa, Roland Bouffanais

**Affiliations:** ^1^ University of Ottawa, Ottawa, ON, Canada; ^2^ Singapore University of Technology and Design, Singapore, Singapore; ^3^ Thales Solutions Asia, Singapore, Singapore

**Keywords:** collective animal behavior, collective decision-making, collective robotics, multi-robot systems, swarm intelligence, self-organization, swarm robotics

## Abstract

In the study of collective animal behavior, researchers usually rely on gathering empirical data from animals in the wild. While the data gathered can be highly accurate, researchers have limited control over both the test environment and the agents under study. Further aggravating the data gathering problem is the fact that empirical studies of animal groups typically involve a large number of conspecifics. In these groups, collective dynamics may occur over long periods of time interspersed with excessively rapid events such as collective evasive maneuvers following a predator’s attack. All these factors stress the steep challenges faced by biologists seeking to uncover the fundamental mechanisms and functions of social organization in a given taxon. Here, we argue that beyond commonly used simulations, experiments with multi-robot systems offer a powerful toolkit to deepen our understanding of various forms of swarming and other social animal organizations. Indeed, the advances in multi-robot systems and swarm robotics over the past decade pave the way for the development of a new hybrid form of scientific investigation of social organization in biology. We believe that by fostering such interdisciplinary research, a feedback loop can be created where agent behaviors designed and tested *in robotico* can assist in identifying hypotheses worth being validated through the observation of animal collectives in nature. In turn, these observations can be used as a novel source of inspiration for even more innovative behaviors in engineered systems, thereby perpetuating the feedback loop.

## 1 Introduction

Social animal groups offer archetypal examples of collective organization and action, whereby repeated local interactions among conspecifics produce emergent dynamic patterns and responses at scales far exceeding the size of the animals involved. Collective animal behavior can be observed over a wide range of spatial scales, spanning from the aggregation of amoeboid cells to the large-scale murmurations displayed by flocks of starlings (Sumpter, 2010). Such collective behaviors are in no way limited to the animal world. Many other disciplines also study the collective actions of what is generally referred to as multi-agent systems, ranging from voter and opinion dynamics models (Proskurnikov and Tempo, 2017; Mateo et al., 2017; Proskurnikov and Tempo, 2018; Redner, 2019) and herding behavior on social networks (Lim and Bouffanais, 2019) in the social sciences, to collective decision-making in the field of swarm robotics (Mateo et al., 2019; Prasetyo et al., 2019; Dorigo et al., 2021). These collective behaviors are the outcome of complex bottom-up dynamic processes involving repeated local interactions of actors evolving in unstructured and dynamic environments filled with stimuli and constraints. Despite the pervasiveness of collective behaviors, a full understanding of the underlying mechanisms that govern the emergence of these complex behaviors is still lacking. As a matter of fact, the study of collective phenomena, and collective behaviors in particular, form a highly active field of multi-disciplinary research (Bouffanais, 2016; Ouellette and Gordon, 2021).

Despite evident common goals across disciplines, biologists often argue that models of collective behavior should be constructed with a particular hypothesis in mind. This hypothesis would then have to be empirically tested and validated for a given species (Ouellette and Gordon, 2021). Indeed, the current literature on collective animal behavior focuses on identifying the underlying motivation or function for such social organization in a given taxon (Halloy et al., 2007; Landgraf et al., 2011; Landgraf et al., 2018; Lei et al., 2020). This overall approach differs from that of physicists and complexity scientists who seek to focus on commonalities in patterns and universal characters exhibited during collective operations by these complex systems. Nonetheless, a more “micro”-approach is always necessary when hunting for a higher level of detail in a given collective behavior (Mitri et al., 2013). When focusing on an individual actor, one can observe a large number of factors, both internal and external, that affect the local interaction rule, thereby guiding that individual’s actions (e.g., stress, desire to mate, desire to conserve energy, etc.) (Katz and Naug, 2015; Li et al., 2021). These behaviors are further confounded when the actions of one individual can also be affected by the actions of other individuals. Ultimately, a full understanding of the collective behaviors observed in nature can only be achieved by inferring the local interaction rules among individuals in a vast range of circumstances. For instance, schooling fish behave differently and may likely use a different set of rules when faced with a threat from a predator (Sosna et al., 2019; Lei et al., 2020). However, unless the behavior of an individual can be controlled, essentially allowing one to isolate the factors that influence that individual’s actions, these local interaction rules can only be described qualitatively (Krause et al., 2011).

Traditionally, collective behaviors have been studied by performing observational studies on animal behavior, thus allowing for the collection of empirical data from animals, as an individual or a collective, in their natural habitat (Radakov, 1973). However, these methods rely heavily on the animal of interest naturally performing the behaviors being studied and the utility of statically placed cameras to record the data (Hughey et al., 2018). This problem is compounded when studying animal collectives as multiple individuals need to be observed, possibly over long periods of time (Berdahl et al., 2018). In addition, the presence, or absence, of certain unknown external stimuli may affect the display of the targeted collective behavior (Rosenthal et al., 2015; Sosna et al., 2019; Sankey et al., 2021). This highlights another key limitations of solely relying on empirically gathered data—the slow rate of data collection (Balch et al., 2006; Halloy et al., 2007; Landgraf et al., 2021).

As such, it can be said that our ability to fully understand collective animal dynamics is hindered by two main challenges: 1) the limited capacity to accurately track the movement of animals and the resultant individual behaviors stemming from the interactions between them (Hughey et al., 2018), and 2) the inability to isolate and study the actions of individual animals that give rise to these collective dynamics, stemming from other internal and external factors that also influence the final behavior of an individual (Katz and Naug, 2015; Li et al., 2021). These limitations have been mitigated to a certain extent by using animals in captivity. Doing so allows for the study of targeted behaviors in a more controlled environment (Rosenthal et al., 2015; Sosna et al., 2019) and also allows experimenters to enjoy a certain degree of control over the collective behaviors displayed (Balch et al., 2006; Krause et al., 2011; Rosenthal et al., 2015; Sosna et al., 2019; Sankey et al., 2021).

However, it can be argued that more can be done to overcome the various challenges posed by the process of collecting empirical collection data. Even with the multi-disciplinary interest in understanding collective behaviors, ideas and hypotheses tend to flow in one direction—from the realm of biology into the fields of physics and robotics. Despite the wealth of insight that can be obtained from the use of alternative methods, such as robotic experiments and physics simulations, these approaches are often met with the argument of low result fidelity; simulations and robotic experiments are often critiqued for being unable to exactly replicate the movement and communication patterns, sensing abilities, behaviors of live animals, as well as the effect of environmental disturbances (Garnier, 2011; Dorigo et al., 2021; Ouellette and Gordon, 2021).

In this paper, we contend that there is a place for such alternative methods in the study of collective animal behaviors. While simulations and models may be relatively simplistic and lack high fidelity results, they afford researchers the ability to control various experiment parameters otherwise constrained by the natural environment. In addition, experiments carried out *in silico* allows for multiple hypotheses to be tested in quick succession, permitting the fast exploration of vast parameter spaces. Hypotheses validated by such virtual simulations can be more thoroughly tested *in robotico*, by means of multi-robot experiments allowing for physical interactions with the environment. We argue that carrying out such experiments using multi-robot systems functions as an intermediary between pure simulations and ethological studies, and is especially important due to the difficulty in accounting for such physical interactions in the virtual world. Naturally, the results ultimately need to be validated through empirically collected data as there may be certain behavioral intricacies that can only be observed in specific animal species. This can initially be done using live animals either within a virtual environment or together with biomimetic robots in mixed societies to retain a certain degree of experimental control before progressing to animal only observations.

**FIGURE 1:**
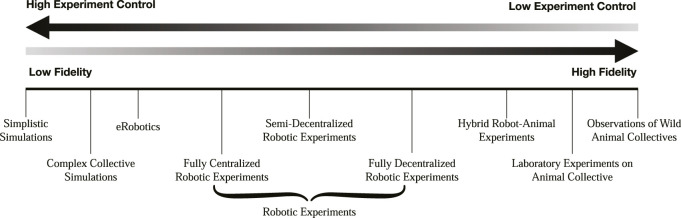
Types of collective behavior experiments. Experiments with models and simulations provide high levels of experimental control while coming at the cost of reduced fidelity. Conversely, performing high fidelity experiments comes at the cost of lower levels of control.

Although simulations have been and are still widely used, we believe that purely robotic experiments continue to be underappreciated and employed too rarely in the study of collective animal behavior. This is despite recent advances in core robotic hardware components and the explosive growth in multi-robot system technology which offers a unique combination of opportunities to expand our research toolkit (Mitri et al., 2013). In this paper, we propose a new workflow allowing for the study of collective animal behavior to be carried out in a quicker manner through a series of tests with increasing levels of fidelity (see Figure 1). Initially, simplistic simulations and models can be used to improve on and rapidly filter out incorrect hypotheses. Subsequently, experiments can be performed using multi-robot systems, allowing for hypotheses to be tested in a physical environment with a high degree of control over the subjects and environment. Hypotheses can be further refined through performing hybrid robot-animal experiments and experiments using animals in captivity before finally validating the most promising ones through the gathering of empirical data of wild animals.

## 2 Simulating Collective Behavior

With the various challenges and difficulties associated with gathering empirical data from the observations of animal collectives in the wild (Hughey et al., 2018; Lei et al., 2020; Li et al., 2021), models and simulations present themselves as quick and easy alternatives to test the viability of hypotheses. Given the long list of questions that biologists wish to have answered, the use of computer simulations will allow for more promising theories to be developed and identified faster, and then later verified with empirical data collection. In addition, these models and simulations are crucial when attempting to predict the collective behavior of animals over many generations (Guttal and Couzin, 2010; Hein et al., 2015; Lei et al., 2020).

The key advantage granted by the use of simulations is one’s ability to identify and isolate a set of parameters that can be tested systematically—a task nearly impossible when dealing with live animal collectives (Eriksson et al., 2010; Krause et al., 2011; Berdahl et al., 2018; Li et al., 2021). This stems from the bottom-up construction of simulations that allow for the precise description of individuals and their interactions with other conspecifics within animal groups (Ouellette and Gordon, 2021). While the identification of these parameters can yield valuable insights into why and how various collective behaviors occur, there is no method of differentiating results of significance from artifacts of the simulations. These simulation artifacts arise from the fact that it is very difficult and computationally expensive to create simulations that take into account all of the complexities of the agents interacting with the physical environment, necessitating the simplification of certain model parameters (Webb, 2001). Small changes in a simulated agent’s behaviors due to these simplifications made may be further amplified in the study of collective behavior, where interactions take place between a large number of individuals and the environment, as well as the interactions between the individuals themselves, leading to the manifestation of such artifacts (Dorigo et al., 2021). As such, there is a critical need to test the validity of parameters in experiments of increasing fidelity (see Figure 1).

The simulations used to validate hypotheses can range from simplistic models, such as the self-propelled particles (SPP) studied by (Vicsek et al., 1995) or the boids developed by (Reynolds, 1987), to the various eRobotics simulation platforms that include more accurate agent dynamics, such as ARGoS (Pinciroli et al., 2012) and ROS (Yan et al., 2017). While testing and experimenting with simple models will cause some loss of accuracy when compared to the data collected from the observation of live animals, such models offer vast amounts of control over the test parameters. Despite the lower level of fidelity, the use of relatively simplistic simulations and mathematical modeling provides a useful tool for a faster iteration cycle than experiments carried out in robots, or observational studies in animals, allowing for the quick rejection of incorrect hypotheses. Indeed, such simple models have helped to explain certain behaviors observed in animal collectives. For example, the SPP model was used by Yates et al. (2009) to show how noise affects the level of coherence in locust swarms and by Polverino et al. (2021) to study the movements of animal groups when threatened by a predator. Similarly, the Ising model was also used by Feinerman et al. (2018) to study the transport of food by ants.

The progression from experimenting with simplistic models to more complex robotic simulations offers the opportunity to test various hypotheses in more realistic scenarios, albeit still with lower fidelity compared to physical robotic experiments and the observation of live animals. This increased realism comes at the price of reduced control over the experiments; robot dynamics need to be accounted for when developing behaviors and the collective behaviors expected from a system may be obscured by noise. One must also be mindful of the available finances and potential logistics challenges when designing such experiments. We believe that many new hypothesis can be found by increased use of systematic experimentation with multi-agent robotic systems in high fidelity environments.

As already mentioned, when testing hypotheses in progressively realistic environments, certain theories may inevitably be proven infeasible or false. However, this does not mean that the research effort has been wasted; these observations can still be re-purposed for applications in computing and engineering. For example, while the agents used by the Particle Swarm Optimization (PSO) algorithm proposed by Kennedy and Eberhart (1995) only mimic the movement patterns of flocks of birds or schools of fish crudely, the PSO algorithms are a popular method for solving certain optimization problems (Wang et al., 2018) and often serve as the starting point for multi-robot systems carrying out target search tasks (Couceiro et al., 2014; Kwa et al., 2020; Kwa et al., 2021).

## 3 Testing Collective Behavior With Robots

Simulations provide a fast and highly controllable way of exploring hypothesis of collective behaviors. However, their lack of fidelity—in terms of modeling the complex physical interactions with the environment and detailed modeling of the agents (Webb, 2001; Li et al., 2020; Dorigo et al., 2021)—may result in various inaccuracies when used to predict the behavior of real-world systems. This is especially true in the case of collective behaviors where the interaction between all the agents plays a large role in the behavior of any individual agent. Here, even small errors in modeling an agents physical interactions with the environment and other agents amplifies the simulation-reality gap greatly in these complex systems (Dorigo et al., 2021).

These limitations can be partially overcome by the use of experiments involving robotic agents. Indeed, there are several examples of robotic platforms built expressly for the purpose of studying animal behavior, such as the SCARAB platform to study collective transport in ants (Berman et al., 2011), the robots developed by Abaid et al. (2012) to study collective motion in zebrafish, and the robotic honeybees used by Landgraf et al. (2011) to study the waggle dance in honeybees. Experiments involving robots can range from purely robotic approaches (Webb, 2001; Fu et al., 2007; Waibel et al., 2009; Gravish and Lauder, 2018; Li et al., 2020) to those that integrate robots with live animals in hybrid robot-biological systems, also known as mixed societies (Halloy et al., 2007; Krause et al., 2011; Halloy et al., 2013; Bierbach et al., 2018; Sankey et al., 2021). As an intermediate between robotic experiments and those involving live animals, certain groups have also employed the use of virtual reality, where animals are exposed to stimuli in a simulated environment to elicit targeted behaviors, allowing researchers to decouple the behavior and the morphology of the stimulus (D’Eath, 1998; Baldauf et al., 2008; Krause et al., 2011; Polverino et al., 2012; Naik et al., 2020). These different experimental methods have different associated levels of control and fidelity (see Figure 1.)

The increase in fidelity when compared to simulations and models stems from the robotic agents’ need to interact with the physical world where they are subject to the disturbances of the environment (e.g., nonuniform friction or soft surfaces to traverse, delays in communications, noisy identification of other agents, directionality of sensors, the movement of other agents etc.). Often, simplifications are made when modeling these disturbances in simulation, which diminish their fidelity at the gain of iteration time. As stated by Hamann, (2018): “Abstract mathematical or computational models as well as simulations may be efficient but have limited credibility and may miss important features of reality.” As such, it is key to clearly identify the intended purpose and limitations of such models and simulations to determine the situations in which they are applicable and when their use is inappropriate (Ouellette and Gordon, 2021). By implementing the collective behaviors in multi-robot systems, many of the physical interactions with the environment—and between agents distributed in space—are integral part of the system, thereby yielding a higher level of fidelity (Gravish and Lauder, 2018; Dorigo et al., 2021).

Furthermore, in comparison to observing live animal behaviors, such multi-robot systems provide experimenters with a much greater level of control over the behavior of the agents and their display of collective behavior. This provides certain benefits over purely behavioral observations that are obtained when observing wild animals, allowing one to quantify the strength and influence of certain interaction rules and behavioral patterns (Romano et al., 2019; Horsevad et al., 2022). It is worth stressing that even without the level of control afforded by multi-robot systems, it would still be possible to infer certain rules and strategies from behavioral observations. However, these inferences are bound to remain at a qualitative and descriptive level (Krause et al., 2011). The added flexibility over pure animal observations, combined with the potential for many more observations, especially of rare events, adds great value to the use of robotic agents in the study of collective behavior. Nonetheless, the level of experimental flexibility does decline when the robotic agents are made to replicate the exact behavior of particular animals, such as in the case of hybrid experiments, where robotic agents interact with real-life animal groups to study the response of the collective (Krause et al., 2011; Sankey et al., 2021).

The increased fidelity of robotic methods compared to that of simulations comes at the cost of longer iteration times when conducting collective behavior experiments with multi-robot systems, for which establishing quantitative and reproducible results can be challenging (Dharmawan et al., 2019; Kit et al., 2019). In addition, experiments involving such multi-robot systems tend to happen on a smaller scale, with physical experiments utilizing a smaller number of agents compared to their virtual counterparts. These problems stem from the significant work needed to maintain such multi-robot testbeds, as well as the financial and logistical challenges faced when designing robotic testbeds and experiments. Furthermore, since the robots are not able to replicate all aspects of the animals themselves, they can lack the agility and sensing abilities of their natural and virtual counterparts, leading to possible discrepancies in the obtained results (Webb, 2001; Kwa et al., 2021).

Besides the challenges associated with longer experimentation times and inconsistent results, hybrid animal-robot experiments with mixed societies and experiments involving virtual reality also face the additional problem of ensuring that the used stimuli trigger the desired response in the targeted group of animals. Despite these difficulties, the conduct of such experiments is still important, especially in the case of gregarious animals and is an aspect of collective behavior that cannot be studied using simulations and mathematical models alone (Romano et al., 2019). In light of these concerns, a large amount of work has gone into studying the different factors that allow a robot to be socially accepted by a group (Abaid et al., 2012; Polverino et al., 2012; Cazenille et al., 2018a; Cazenille et al., 2018b; Li et al., 2021). Similar work has also been done to investigate an animal’s response to stimuli in virtual environments (Baldauf et al., 2008; Stowers et al., 2017; Naik et al., 2020). However, this problem also presents an opportunity to study the cues that trigger certain behaviors and determine what affects the strength of social interactions. Depending on the species studied, an animal’s behavior can be triggered through a stimulus’ visual appearance or movement patterns (Abaid et al., 2012; Marras and Porfiri, 2012; Li et al., 2021), pheromones (Halloy et al., 2007), or physical touch (Anstey et al., 2009).

While there are conflicting goals in the way different disciplines study collective behaviors (Ouellette and Gordon, 2021), having collective behaviors embodied in multi-robot systems provides knowledge of what is possible with different types of behavior. While there are many examples of this in the literature, here we only mention a few examples on different uses of robots in the study of collective behavior. In Vallegra et al. (2018) and Zoss et al. (2018), a heterogeneous autonomous buoy system was used to perform experiments in a physical environment. These were, among other things, used to study how the amount of connectivity affects the response of the collective system in order to find theoretical reasons why many animal groups operate with a limited connectivity or modulate it depending on different factors (Attanasi et al., 2014). The embodiment of the agents in physical space makes the analytical findings more robust due to the physical interactions with the environment. High fidelity robots that mimicked fish were used in (Li et al., 2020; Li et al., 2021) to study how fish position themselves while swimming to exploit the generation of vortices. In their article, the authors commented on the challenges of carrying out experiments with live fish schools and the difficulty in replicating the complex hydrodynamics and relevant Reynolds numbers while using computational simulations (Li et al., 2021). This indicates that an approach with robots was uniquely suited to study this particular problem. High fidelity robots were also recently employed in (Sankey et al., 2021) where a drone, disguised as a falcon, was used to induce a response in a flock of pigeons when close to a predator. This was used to test the selfish herd hypothesis in pigeons—of whether the pigeons will flock together for selfish protection or align to flee faster—for which ambiguous results were found using a purely simulation and modeling approach. In Krause et al. (2011) hybrid experiments are also described, with a controlled fish being used to induce a fleeing response in a school of fish and of robots that can integrate into groups of cockroaches and be used to steer their collective behavior. In addition to these examples, there are many other possibilities in the usage of robotics in the study of collective behaviors, with many considerations for particular problems (Webb, 2001; Halloy et al., 2007; Gravish and Lauder, 2018; Ouellette and Gordon, 2021). The incorporation of such multi-robot experiments as a staple into the exploration of collective behavior hypotheses is bound to bring value to the endeavor, especially when ideas flow in both directions between robotics and biology.

## 4 Discussion

In the quest to fully understand the underlying mechanisms that govern collective animal behavior, many researchers rely on the tried and tested method of gathering empirical data in observational studies of animal collectives in the wild. However, due to the limitations of current technology and the difficulty in observing the targeted set of behaviors, the collection of such observational data happens slowly and can be a time consuming process. To avoid this, physicists and complexity scientists rely on abstract simulations that model the emergent collective behaviors stemming from the repeated interactions between individuals. Powerful abstraction such as complex network theory are also considered to analyze the collective as a superorganism at the system level (Sekunda et al., 2016). Despite the lack of specificity in such models, commonalities in patterns and universal characters exhibited by these complex systems collective operations can be gleaned from the results. This can be done thanks to the highly controlled nature of the simulations, allowing the key variables that influence the targeted behavior to be rapidly pinpointed. In addition, due to such forms of experimentation being virtual, multiple theories and hypotheses can be tested and validated in quick succession.

The use of robotics stands at the intersection between the gathering of empirical data and abstract modeling and can serve as a promising intermediary between the two. While the development and validation of theories with robotic experiments will naturally have a longer iteration time compared to models and simulations, testing collective behavior hypotheses in such a manner will result in an increase in the fidelity of the data obtained. This is due to the fact that robotic experiments allow for an individual’s interaction with the physical environment to be more realistically considered in addition to the multiple interactions between agents. Of course care should be taken to ensure the robots employed capture the specific dynamics under consideration in a sufficient capacity. As such, the use of robot systems allows for theories and hypotheses developed in virtual simulations to be validated in a controlled physical setting before being further confirmed in live animals. Even when theories are proven to be false through the process of validation, they may still be found useful and serve as the inspiration for the approaches used by roboticists and artificial intelligence practitioners. For example, based on the simple physics models developed by Berman et al. (2011) and Rubenstein et al. (2013), robotic tests were carried out by Wilson et al. (2018) showing that the maximum transport speed of a group of ants is based on the maximum speed of the slowest group member. These results were then validated by correlating them with observations of live ants carried out by Buffin et al. (2018). The initial findings by Berman et al. (2011) and Rubenstein et al. (2013) also served as inspiration for Christensen et al. (2016), who developed a robot platform capable of collectively pulling large loads.

The use of robotic experiments to explore collective behaviors does not need to happen in purely engineered multi-robot systems. Biomimetic robots can be used to trigger targeted behaviors in hybrid robot-animal experiments. In addition to developing robots that mimic the morphology and behaviors of the targeted animal, intra-species interactions can also be studied. For example Sankey et al. (2021), carried out an experiment involving live pigeons together with a falcon UAV, allowing them to demonstrate that pigeons turn away from the flock when in the vicinity of a predator. With such hybrid experiments, the study of collective behaviors move from being a mainly descriptive endeavor, to have the ability to manipulate the collective behavior and gain some degree of control over the experiment, and being able to test specific hypotheses (Krause et al., 2011). Besides the triggering of targeted behaviors, animal mimicking robots can also be used for data gathering. Such robots can be embedded within animal groups, facilitating more accurate data collection of the animal collective such as accurate GPS position information or detailed postural information of different individuals. Gathering such information could permit a better quantification of behavioral states and enhance the understanding of how social interactions affect these collective behaviors (Krause et al., 2011; Halloy et al., 2013; Hughey et al., 2018). In this era of explosive growth of deep learning methods, such vast troves of data can also be further used to feed artificial neural network methods aimed at identifying “hidden” correlations and patterns. Given their need for very large data sets, the full power of these deep learning methods cannot be harnessed with sparse empirical data sets.

Currently, observations made by biologists often serve as the inspiration for the strategies developed by roboticists (Dharmawan et al., 2019), while the reverse flow of ideas is significantly smaller. This one-way flow of ideas still has the potential to be extended to smaller scales—in relation with self-organization at the micro- or nano-scales by active matter, cells and bacteria—owing to recent progress in miniaturization. However, we believe that closer multi-disciplinary research between these two fields will yield benefits and many noteworthy developments to members of both communities. Recently Gravish and Lauder (2018), coined the phrase robotics-inspired biology, where discoveries made in the realm of robotics have served to develop theories and hypotheses to be validated with wild animal observations. With the increasing interest and accessibility of multi-robot systems (Dorigo et al., 2021), it is our hope that such interdisciplinary research can be fostered, leading to the creation of a similar feedback loop where observations in nature can be used as the starting point for strategies used by collective robot systems. Human-designed agent behaviors can then serve as a point from which hypotheses can be generated for validation in biological systems, thereby perpetuating, and possibly amplifying the feedback loop.

## Data Availability

The original contributions presented in the study are included in the article/supplementary material, further inquiries can be directed to the corresponding author.
